# Influence of Creep Damage on the Fatigue Life of P91 Steel

**DOI:** 10.3390/ma15144917

**Published:** 2022-07-14

**Authors:** Stanisław Mroziński, Zbigniew Lis, Halina Egner

**Affiliations:** 1 Faculty of Mechanical Engineering, Bydgosz University of Science and Technology, Al. Prof. S. Kaliskiego 7, 85-796 Bydgoszcz, Poland; stmpkm@pbs.edu.pl (S.M.); zbigniew.lis@pbs.edu.pl (Z.L.); 2 Faculty of Mechanical Engineering, Cracow University of Technology, Al. Jana Pawła II 37, 31-864 Kraków, Poland

**Keywords:** low-cycle fatigue, creep, damage, strain energy

## Abstract

The following paper presents the results of tests on samples made of P91 steel under the conditions of simultaneously occurring fatigue and creep at a temperature of 600 °C. The load program consisted of symmetrical fatigue cycles with tensile dwell times to introduce creep. Static load (creep) was carried out by stopping the alternating load at the maximum value of the alternating stress. The tests were carried out for two load dwell times, 5 s and 30 s. A comparative analysis of the test results of fatigue load with a dwell time on each cycle confirmed that creep accompanying the variable load causes a significant reduction in sample durability. It was shown in the paper that regarding the creep influence in the linear fatigue damage summation approach, it is possible to improve the compliance of the fatigue life predictions with the experimental results.

## 1. Introduction

Analysis of the operational loads of many structural elements indicates that they are often subject to mechanical loads where the independent quantity is force (e.g., pressure) and the dependent quantity is the deformation of the element. This applies, inter alia, to facilities operating at elevated temperatures. This type of load may be additionally accompanied by material creep, which changes the nature of the load and the durability of the designed elements. Creep is much more pronounced at high temperatures, for example, in pipelines that contain a pressurized hot medium or gas turbine components that are statically loaded but operate at high and variable temperatures. In such cases, when predicting the fatigue life of a technical object, only the fatigue characteristics are taken into account, obtained for example, under the conditions of controlled deformation ($\varepsilon_{ac}$ = const) or stresses ($\sigma_{a}$ = const), while in real applications, the conditions are different, which may lead to divergent results obtained from calculations and tests. In papers [1,2,3,4], the results of low-cycle fatigue tests are presented for samples made from the same material, obtained in the conditions of $\sigma_{a}$ = const and $\varepsilon_{ac}$ = const. Based on the comparative analysis of the fatigue diagrams in the $2N_{f}-\varepsilon_{ac}$ coordinate system, it was found that at the same deformation levels, the fatigue life under the conditions $\sigma_{a}$ = const is lower than that obtained in the conditions of controlled deformation ($\varepsilon_{ac}$ = const). The deformation asymmetry during the half-cycles of tension and compression, which caused the cyclic creep of the material, was given as the reason for the reduction in durability under the conditions $\sigma_{a}$ = const. Creep-fatigue experiments were conducted in [5] under stress-controlled conditions to understand dwell time’s effects on the durability of a DS superalloy. It was shown that the most important damage source is creep-fatigue interaction damage which is controlled by load amplitude, stress ratio, and dwell time per cycle. In paper [6], multiaxial creep-fatigue life and its variations with the increasing holding time under different loading programs were investigated for 304 stainless steel. The introductions of holding periods under creep-fatigue loadings resulted in lifetime reductions as compared with pure fatigue loadings. The problem of creep-fatigue interaction for various engineering materials was also recently investigated in [7,8,9]. In papers [10,11], tests were carried out to determine the effect of the sequence of fatigue cycles and static loads in the load program. It was found that the sequence of both loads in the load program affects the durability. Variable loads preceding static loads result in obtaining higher durability than the variant in which the static load precedes the variable loads. In light of the obtained results, it was found that creep damage and fatigue damage are not independent. These results confirm the observations included, inter alia, in [12], where the authors found that short cracks appear during the variable load, and their density is larger if the material is initially subjected to a static load. In paper [10], experimental verification of the linear hypothesis of damage summation [13] was also carried out. Since the linear summation hypothesis is insensitive to the sequence of events in the load program, it may provide erroneous results of the durability assessments in comparison to the experimental data.

The problem of fatigue life predictions under the conditions of simultaneous occurrence of the variable and the static load was analyzed with the use of various fatigue descriptions, i.e., deformation, stress, or energy approach. In the works [11,14,15,16,17,18,19,20], the problem of material fatigue was attempted to be explained with the use of energy description. The results indicate significant advantages of the energy approach, even if a relatively simple combination of the static and variable load effects was applied. The main advantage of the energy description is the ability to accumulate damage using plastic strain energy as a criterion parameter.

In papers [21,22,23], the authors investigated the influence of creep on fatigue life and verified currently used calculation models. Several modifications of the classic Palmgren-Miner’s linear fatigue damage summation hypothesis [13] were proposed. The authors concluded that disregarding creep damage in the calculations may lead to a significant differentiation in comparison with the test results. The creep duration influences the results diversion from the experiment.

The present work is a continuation of research on the improvement of constitutive modeling of low cycle fatigue (cf. [1,4,11]), as well as on the explanation of the phenomena accompanying the load program containing constant and variable loads.

## 2. Materials and Methods

### Experimental Testing

Samples for fatigue tests were prepared from P91 steel (*R_m_* = 716 MPa, *R_e_* = 564 MPa, *A*_5_ = 35% at room temperature). The chemical composition of P91 steel is shown in Table 1.

**Figure 1:**
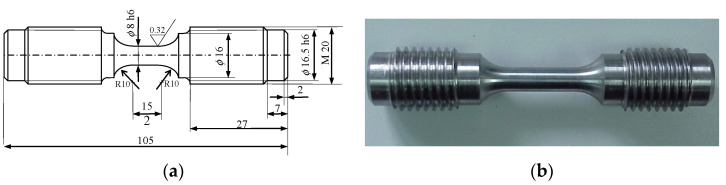
(**a**) Test sample dimensions (in [mm]); (**b**) test sample view.

The samples were cut out from a thick-walled pipe for power industry applications, with an external diameter of 200 mm and a wall thickness of 20 mm. The samples were shaped under the guidelines specified in the standard [24]. The dimensions of the sample are shown in Figure 1.

**Figure 2:**
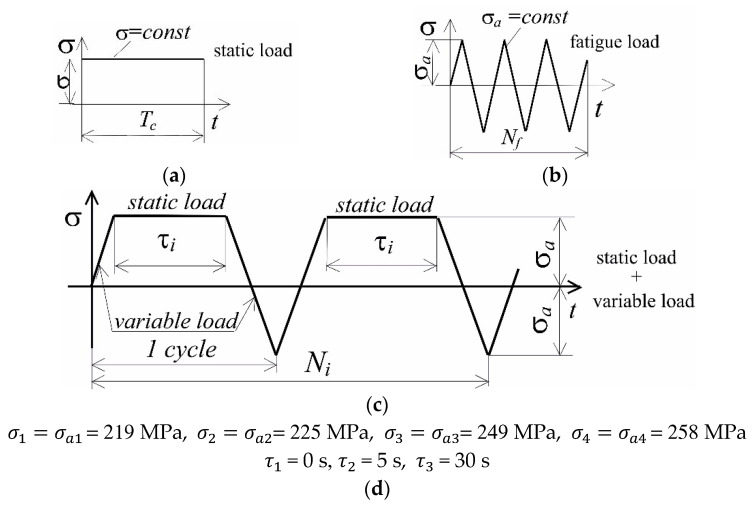
Load programs: (**a**) static load, (**b**) fatigue load, (**c**) static load + fatigue load, (**d**) load parameters.

The load program of experimental tests included: static tensile tests, creep tests, low-cycle fatigue tests, and tests in which the samples were subjected to fatigue with a dwell time in each cycle. The load programs and their symbols are shown in Figure 2.

**Figure 3:**
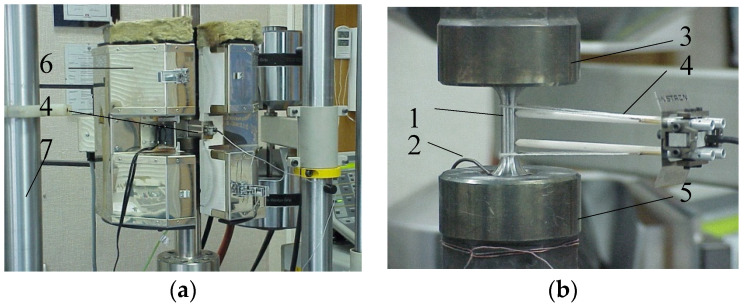
Test stand: (**a**) heating chamber; (**b**) sample in grips. 1—sample, 2—thermocouple, 3—upper grip, 4—extensometer, 5—lower grip, 6—heating chamber, 7—machine frame.

**Figure 4:**
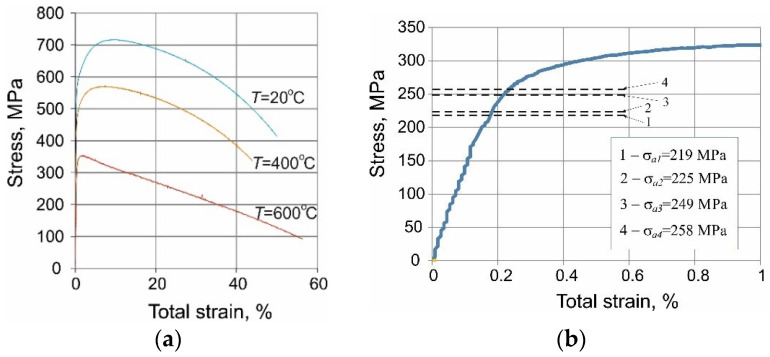
Monotonic stress–strain curves: (**a**) for different test temperatures, (**b**) stress amplitude levels chosen for creep-fatigue tests (*T* = 600 °C).

Experimental tests (static and fatigue tests) were carried out at a temperature of *T* = 600 °C on an Instron 8502 testing machine, equipped with a heating chamber with a maximum temperature range of 1000 °C (Figure 3). The temperature of the sample was monitored with a thermocouple attached to the sample. The sample deformation was measured with an extensometer with a measurement base of 12.5 mm. Fatigue tests in the conditions $\sigma_{a}$ = const were carried out on four stress levels (see Figure 2d), determined based on the analysis of static tension diagrams (Figure 4b). The load frequency was 0.2 Hz. During the tests, the instantaneous values of the force loading the sample and its deformation were recorded. Two fatigue tests were performed at each load level.

## 3. Results

### 3.1. Static Tension Test

Figure 4a present a stress–strain curve obtained at temperature T = 600 °C. To illustrate the influence of temperature on strength properties, the figure additionally includes the tensile curves of P91 steel obtained at two other temperatures, 20 °C and 400 °C. The stress in the tested sample was calculated as the ratio of the instantaneous force loading the sample and the initial cross-sectional area of the sample (nominal stress). Figure 4b include a fragment of the tensile diagram obtained at the temperature T = 600 °C, with the stress amplitude levels for the creep-fatigue tests indicated in it, see Figure 2d.

The tensile diagrams (Figure 4) were subjected to a detailed analysis aimed at determining the basic strength parameters (see Table 2).

The comparative analysis of the data in Table 2 confirm the common literature reports on the influence of temperature on the basic strength parameters of P91 steel [25,26,27,28]. Based on the analysis of the tensile diagrams (Figure 4) and the values of the strength parameters listed in Table 2, it can be concluded that the increase in temperature leads to a reduction in the yield point ($R_{p0.2}$). A similar relationship is observed in the case of the tensile strength ($R_{m}$) and Young’s modulus (E), for which a decrease in this parameter is also visible. The decrease in strength properties with temperature is accompanied by an increase in elongation ($A_{12.5}$) and constriction (Z).

### 3.2. Fatigue Tests

**Figure 5:**
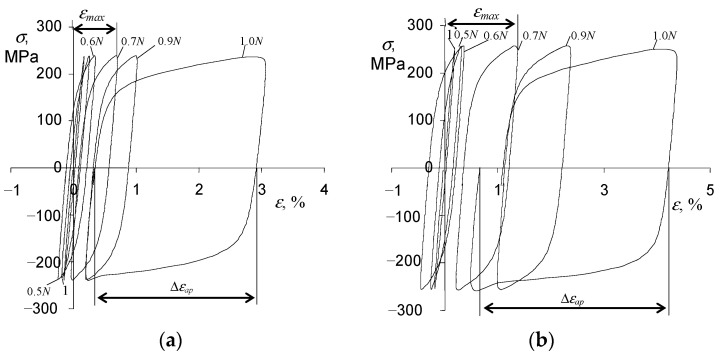
Hysteresis loops recorded in fatigue tests (τ = 0, T = 600 °C): (**a**) $\sigma_{a}$ = 225 MPa, (**b**) $\sigma_{a}$ = 249 MPa.

The analysis of the fatigue test results was carried out using the hysteresis loop parameters ($\sigma_{a}$, $\varepsilon_{ap}$, $\varepsilon_{ac}$), which are necessary for the analytical description of the cyclic properties of steel following the standard [24]. Changes in the hysteresis loop parameters in function of the number of a load cycle were observed during all the fatigue tests. As expected, cyclic creep of the sample material was observed under the conditions of controlled stress ($\sigma_{a}$ = const). The phenomenon consisted of the shift of the hysteresis loop along the strain axis, increasing the maximum strain on a cycle $\varepsilon_{max}$. Figure 5 show exemplary hysteresis loops at two stress levels ($\sigma_{a}$ = 225 MPa, $\sigma_{a}$ = 249 MPa). The presented loops refer to a load program in which creep time τ=0 (see Figure 2).

**Figure 6:**
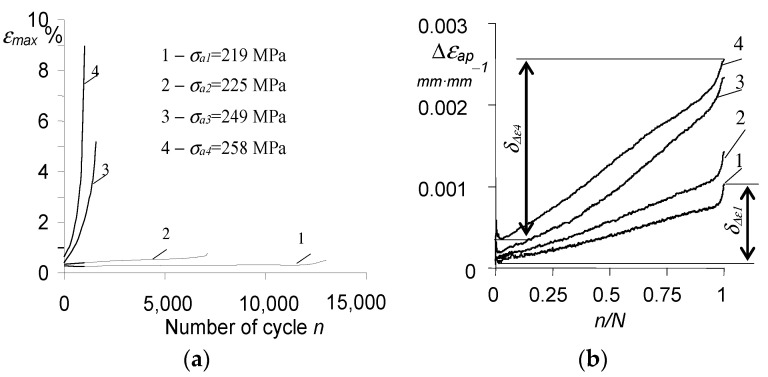
Evolution of: (**a**) maximum strain on cycle; (**b**) plastic strain on cycle $\Delta \varepsilon_{ap}$.

Based on the hysteresis loops analysis, it can be concluded that the amount of maximum strain on a cycle ($\varepsilon_{max}$) during the fatigue test is influenced by the stress level $\sigma_{a}$. As expected, this influence is the smallest at the lowest stress levels and increases with increasing stress. It can also be seen that the strain increments are characterized by different rates, low at the beginning of the test and increasing with the number of a load cycle (Figure 6a).

.

Based on the analysis of the loops shown in Figure 5, it can be concluded that with the increase in the number of the load cycle, the range of plastic deformation $\Delta \varepsilon_{ap}$ also increases at a growing rate. The amount of plastic deformation in a cycle, $\Delta \varepsilon_{ap},$ is also influenced by the stress level $\sigma_{a}$. To illustrate this influence, Figure 6b summarize the changes of $\Delta \varepsilon_{ap}$ in function of a load cycle number n related to the fatigue life N.

The observed increase in the range of plastic deformation proves a clear cyclic softening of the P91 steel at the temperature of 600 °C. To quantify the softening, a coefficient $\delta_{\Delta \varepsilon }$ was introduced (see Figure 6b), defined by the following relationship:

(1) $$\delta_{\Delta \varepsilon }=\Delta \varepsilon_{ap\left(N\right)}-\Delta \varepsilon_{ap\left(1\right)}$$

where $\Delta \varepsilon_{ap\left(1\right)}$ is the range of plastic deformation in the first cycle, while $\Delta \varepsilon_{ap\left(N\right)}$ is the range in the last cycle. It can be seen in Figure 6b that with the increase in stress amplitude, the value of the coefficient $\delta_{\Delta \varepsilon }$ also increases ($\delta_{\Delta \varepsilon 4}$ > $\delta_{\Delta \varepsilon 3}$ > $\delta_{\Delta \varepsilon 2}$ > $\delta_{\Delta \varepsilon 1}$).

### 3.3. Creep Tests

**Figure 7:**
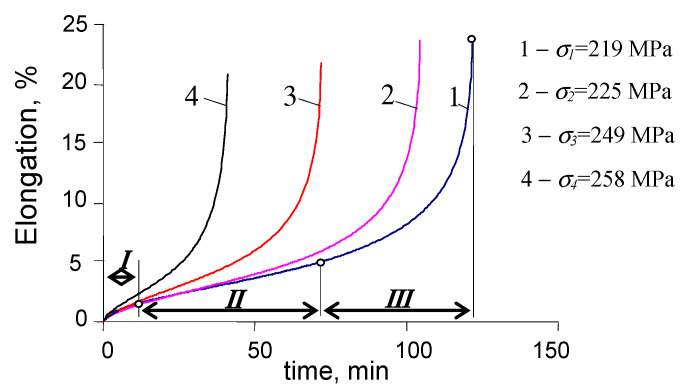
Sample elongation in creep test.

During the creep tests, similarly to the fatigue tests, the instantaneous values of the sample elongation as a function of the creep test time were recorded. As expected, the results of the creep tests are influenced by the constant stress level σ. Figure 7 present the sample elongation vs. time recorded during the creep tests at four stress levels (cf. also [11]).

All creep-to-fracture curves exhibit three stages with different elongation rates of the specimen over time. These stages were indicated on the creep curve obtained for the lowest stress ($\sigma_{1}$ = 219 MPa), stage I with a variable elongation rate, stage II with a constant rate, and stage III also with a variable creep rate. The stress level σ affects both the length of these stages as well as the creep rate in the individual stages.

### 3.4. Fatigue-Creep Tests

**Figure 8:**
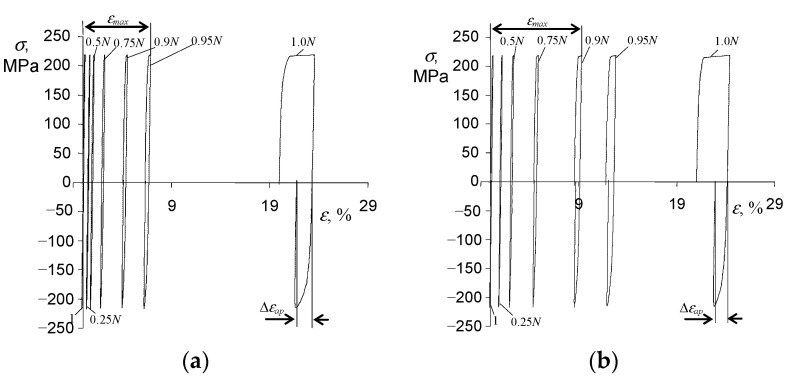
Hysteresis loops in creep-fatigue conditions for $\sigma_{a}$ = 219 MPa, and (**a**) τ = 5 s, (**b**) τ = 30 s.

**Figure 9:**
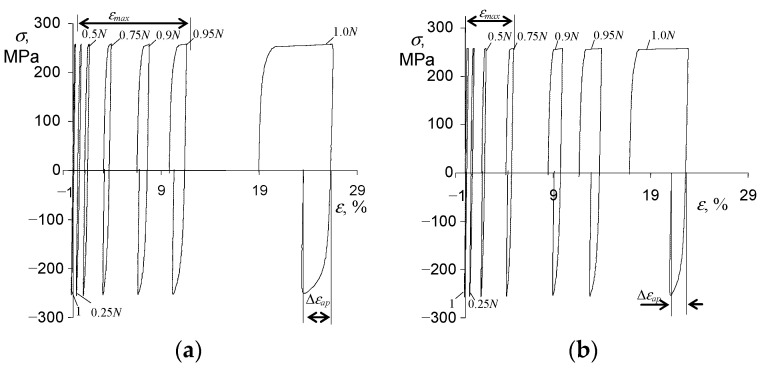
Hysteresis loops in creep-fatigue conditions for $\sigma_{a}$ = 258 MPa, and (**a**) τ = 5 s, (**b**) τ = 30 s.

As expected, during the programs containing both variable and static loads (τ>0, see Figure 2c), cyclic creep of the material was observed. It manifested in the horizontal shift of the hysteresis loops and the increase in the maximum strain on a cycle $\varepsilon_{max}$. Figure 8 and Figure 9 present chosen hysteresis loop positions at two stress levels, $\sigma_{a}$ = 219 MPa and $\sigma_{a}$ = 258 MPa.

**Figure 10:**
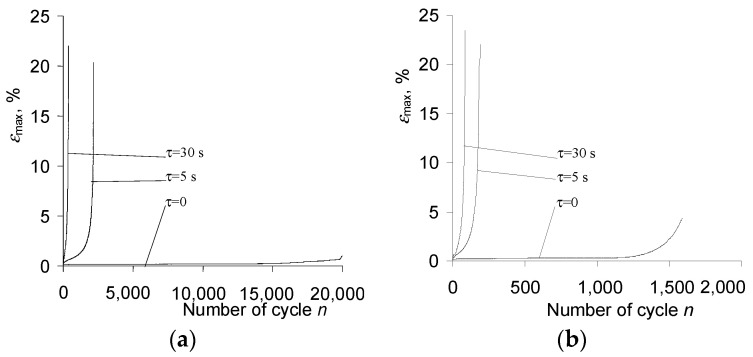
Maximum strain on cycle $\varepsilon_{max}$ vs. number of cycles for different dwell times τ and different stress amplitudes: (**a**) $\sigma_{a}$ = 219 MPa, (**b**) $\sigma_{a}$ = 258 MPa.

It can be seen from Figure 8 and Figure 9 that the creep time affects both the maximum strain $\varepsilon_{max}$ and the range of plastic deformation in a cycle. Figure 10 shows the maximum strains at two stress levels and three values of the dwell time τ.

**Figure 11:**
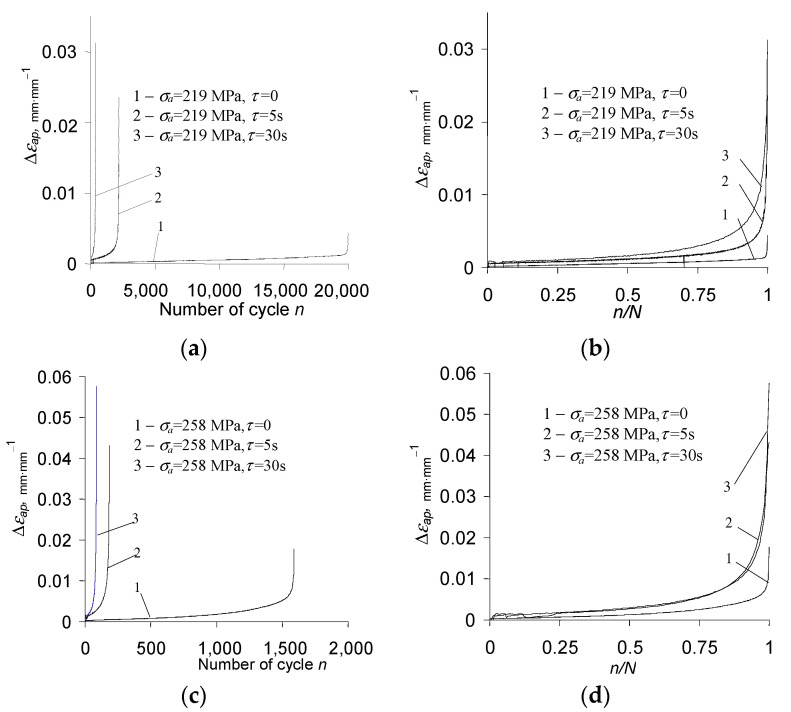
Changes in plastic strain on cycle $\Delta \varepsilon_{ap}$ for different stress amplitude levels: (**a**,**b**) $\sigma_{a}$ = 219 MPa; (**c**,**d**) $\sigma_{a}$ = 258 MPa.

On the basis of the above results (Figure 8 and Figure 9), it can be concluded that creep occurring during the variable load (τ>0) causes a significant increase in the maximum strain $\varepsilon_{max}$. With the increase in a dwell time, the elongation of the sample in the fatigue test increases significantly. The analysis of the position of successive hysteresis loops (Figure 8 and Figure 9) in the function of the number of a cycle allows concluding that under the conditions of combined variable and constant load, P91 steel does not exhibit a stabilization of its cyclic properties. Regardless of the level of alternating stress amplitude $\sigma_{a}$*_,_* and the duration τ of the permanent load, the steel always exhibits significant cyclic softening. This is illustrated in Figure 11 by the increase in plastic strain $\Delta \varepsilon_{ap}$ as a function of the number of a load cycle for various test conditions. In order to compare changes in plastic strains for different dwell times τ, Figure 11b,d present results in the function of relative durability n/N.

Based on the analysis of changes in the plastic strain range $\Delta \varepsilon_{ap},$ it can be concluded that the magnitude of changes in this parameter is influenced, among others, by the dwell time τ. At the same amplitude stress levels, under the conditions of combined fatigue and creep loads, plastic strains $\Delta \varepsilon_{ap}$ are larger than under the conditions of pure fatigue $\sigma_{a}$ = const ($\delta_{\varepsilon 2}>\delta_{\varepsilon 1}$, see also Figure 6b).

**Figure 12:**
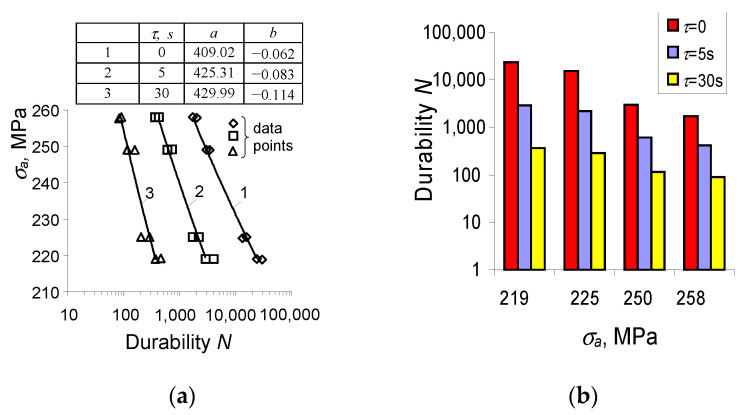
Durability results (experiment): (**a**) fatigue curves, (**b**) summary of durability results.

During the experimental tests, a very clear effect on the fatigue life of the dwell time τ was observed. The fatigue life experimental results obtained for various load program sequences are summarized in Figure 12 in the form of fatigue diagrams. The durability results at four stress levels were approximated in the semi-logarithmic coordinate system by a regression equation of the form:

(2) $$\sigma_{a}=a\log N+b$$

It can be observed that the creep time τ significantly affects the fatigue life. With the increase of time τ, the fatigue life decreases. The results confirm the research described in [21,22,23].

### 3.5. Fractographic Observations

**Figure 13:**
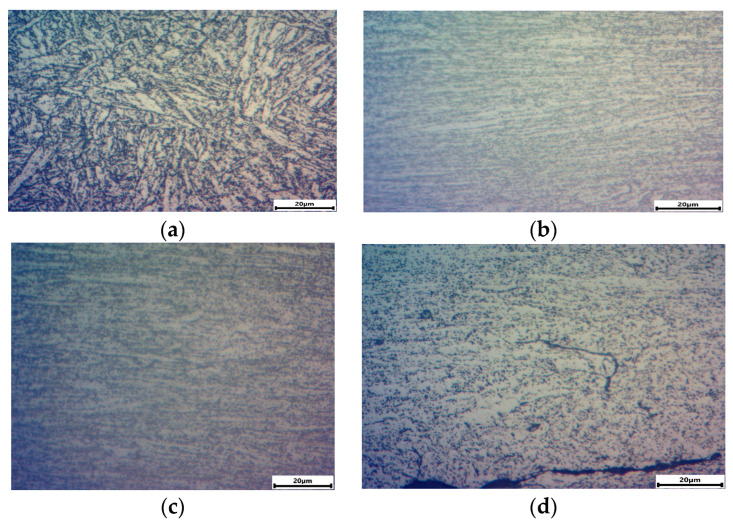
P91 steel microstructure: (**a**) the initial material, (**b**) after uniaxial tension test (T = 600 °C), (**c**) after creep test (T = 600°C, σ = 258 MPa), (**d**) after fatigue test (T = 600 °C, $\sigma_{a}$ = 258 MPa).

After testing, the samples were subjected to fractographic analysis. The fractographic samples were prepared from the measurement parts of the samples, parallel to the load direction. Figure 13 show the sample surfaces after selected variants of the load program. For comparison, the surface of the P91 steel sample before the test is also included in Figure 13a.

P91 steel (Figure 13a) is characterized by a typical microstructure for the class of steels containing 8 ÷ 12% Cr; the microstructure of highly tempered martensite with numerous precipitates of carbides and nitrides is observed. A lamellar microstructure of tempered martensite with numerous precipitates visible at the boundaries of the former austenite grains and martensite lamellae are visible. After the tensile test at *T* = 600 °C (Figure 13b), as expected, a highly deformed microstructure with visible banding is detected—the direction of a material flow corresponds to the direction of the principal stress. Numerous precipitations are visible on the deformation bands.

A similar structure as after the tensile test was observed after the creep test (Figure 13c). The visible structure is also lamellar, with numerous precipitates. 

The structure of the P91 steel after the fatigue test is also characterized by lamellar microstructure (Figure 13d); however, the grain deformation is less pronounced than in the creep test or monotonic test. The deformed microstructure shows the process of coagulation of the precipitates. The main crack propagates transcrystalline, while the secondary cracks are formed along the grain boundaries of the former austenite.

The results of microscopic analyses confirm the necessity regard creep damage during the fatigue life calculations. However, the determination of the quantitative impact of constant and variable load on changes in the microstructure requires further extensive research program.

### 3.6. Durability Assessments

Currently, there are several hypotheses for summing the fatigue damage that evolves under the conditions of simultaneously occurring fatigue and creep loads. These were discussed, inter alia, in [14]. In the present research, a linear model was used for durability calculations, which refers directly to the approach proposed in [13]. For the load program shown in Figure 2, the total damage $D_{t}$ will be equal to the sum of fatigue damage $D_{f}$ and creep damage $D_{c}$:

(3) $$D_{f}+D_{c}=D_{t}.$$

The experimental verification of the linear damage summation model under the conditions of simultaneous creep and fatigue was then carried out. After realizing n cycles of variable load with additional creep ($\tau_{i}>0$) in each load cycle, the total damage $D_{t}$ will be equal to:

(4) $$\sum \frac{n}{N}+\sum \frac{\tau }{T_{c}}=D_{t}$$

where N is the number of cycles to failure in pure fatigue ($\sigma_{a}$ = const and τ=0), while $T_{c}$ denotes the total lifetime in creep (σ = const). It should be emphasized that the approach described by Equation (4) is insensitive to the order of occurrence of particular types of load and ignores changes in the microstructure of the material under various load conditions. This problem was discussed, inter alia, in papers [10,11].

**Figure 14:**
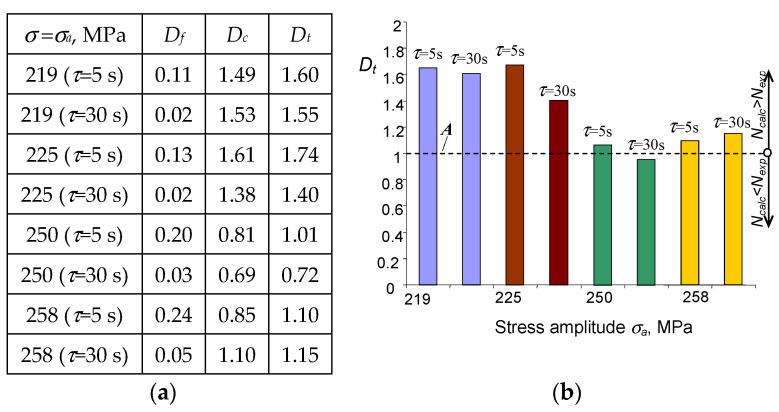
(**a**) Damage levels resulting from calculations; (**b**) interpretation of results.

The results of calculations of damages $D_{f}$, $D_{c}$, and $D_{t}$ are summarized in Figure 14.

**Figure 15:**
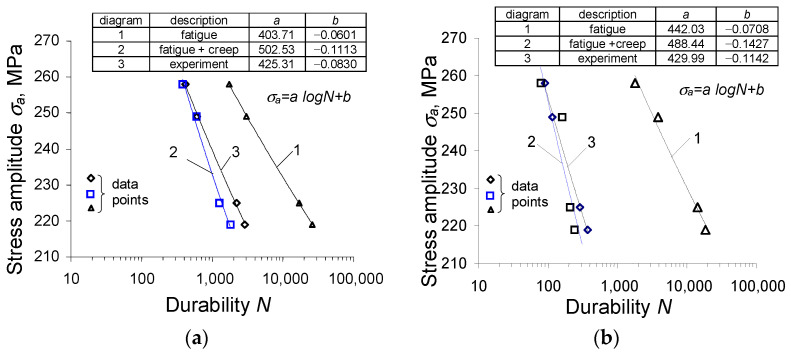
Durability diagrams (experimental and calculated): (**a**) τ=5 s, (**b**) τ = 30 s.

Based on the comparative analysis of the values of the total damage components $D_{t}$ (composed of $D_{f}$ and $D_{c}$), it can be concluded that the dominant component is the creep damage, $D_{c}$. The application of the linear model of damage summation under the conditions of simultaneous occurrence of static load (creep) and variable load (fatigue) results in obtaining total damage larger than unity ($D_{t}>1$) for most of the analyzed load program sequences. Consequently, the calculated durability is lower than that obtained from the tests. This conclusion is confirmed in Figure 15, presenting durability diagrams obtained from calculations and experimental tests. The figure includes the following graphs:

(1) a calculation-based diagram taking into account only the fatigue properties (fatigue);
(2) a calculation-based diagram regarding fatigue and creep (fatigue + creep);
(3) a diagram obtained based on experimental results (experiment).

Analysis of the results presented in Figure 15a,b indicates that regarding creep during the fatigue life calculations significantly improves the efficiency of the durability assessments. If both the creep and fatigue damage is considered within the classical damage summation approach (4), the predicted durability calculations are lower but close to the results of durability obtained from the experimental tests. If only the fatigue damage is considered, the simulated durability is much larger than the real one observed in the experiment.

## 4. Conclusions

During fatigue tests on P91 steel samples at the temperature of 600 °C under alternating variable and constant loads, cyclic softening of the material was observed, both for the programs containing only the pure fatigue load cycles and for the cases when alternating fatigue and creep took place. The amount of P91 steel softening is influenced by both the load level and the dwell time (creep). With the increase of the dwell time in the cycles, the cyclic softening becomes more pronounced.

The increase in the static load time ($\tau_{i}>0$) causes a marked increase in the cyclic creep observed during classic fatigue tests conducted under the conditions of $\sigma_{a}$ = const ($\tau_{i}=0$), and in the range of inelastic stain on a cycle $\Delta \varepsilon_{ap}$.

The dwell periods occurring during the variable load reduce the fatigue life. The dwell time value influences the durability.

Fatigue life predictions that do not regard the material damage due to creep may lead to a significant overestimation of durability. Taking into account the creep damage in the calculations improves the compliance of the numerical and experimental results.

## Figures and Tables

**Table 1:** Chemical composition of P91 steel.

C	Si	Mn	P	S	Cr	Mo	Ni
0.197	0.442	0.489	0.017	0.005	8.82	0.971	0.307
Al	Co	Cu	Nb	Ti	V	W	
0.012	0.017	0.036	0.074	0.004	0.201	0.02	

**Table 2:** Strength parameters of P91 steel at different temperatures.

*T*, °C	*R_p_*_0.2_, MPa	*R_m_*, MPa	*Z*, %	*A*_12.5_, %	*E*, MPa
20	564	716	62	35	209,850
400	463	571	69	44	183,220
600	317	353	72	56	132,890

## Data Availability

Not applicable.
